# Congenital Bands with Intestinal Malrotation after Propylthiouracil Exposure in Early Pregnancy

**DOI:** 10.1155/2015/789762

**Published:** 2015-11-18

**Authors:** Alexander A. Leung, Jennifer Yamamoto, Paola Luca, Paul Beaudry, Julie McKeen

**Affiliations:** ^1^Division of Endocrinology and Metabolism, Department of Medicine, University of Calgary, Calgary, AB, Canada T2T 5C7; ^2^Division of Endocrinology and Metabolism, Department of Pediatrics, University of Calgary, Calgary, AB, Canada T3B 6A9; ^3^Division of Pediatric Surgery, Department of Surgery, University of Calgary, Calgary, AB, Canada T3B 6A9

## Abstract

Exposure to propylthiouracil in early pregnancy may be associated with an increased risk of birth defects. But the spectrum of associated congenital anomalies is not yet well defined. While preliminary reports suggest that most cases of propylthiouracil-associated birth defects are restricted to the preauricular and urinary systems, careful consideration should be given to other possible manifestations of teratogenicity. We propose that congenital bands may potentially represent a rare yet serious complication of propylthiouracil exposure in early pregnancy, possibly arising from an early mesenteric developmental anomaly. We report a case of a 17-day-old girl that presented with acute small bowel obstruction associated with intestinal malrotation arising from several anomalous congenital bands. Her mother was treated for Graves' disease during pregnancy with first trimester exposure to propylthiouracil but remained clinically and biochemically euthyroid at conception and throughout the duration of pregnancy. This case suggests that the use of propylthiouracil in early pregnancy may be associated with congenital bands and intestinal malrotation. More reports are needed to further support this association.

## 1. Introduction

Antithyroid drugs, such as propylthiouracil (PTU) and methimazole (MMI), are widely considered to be first line therapy in the treatment of hyperthyroidism in pregnancy [1–3]. Still, treatment is not without potential risk. The use of MMI in pregnancy has been associated with an increased risk of congenital anomalies [4–6]. PTU, on the other hand, may rarely cause severe maternal hepatotoxicity [3, 7]. In order to balance these risks, a number of expert panels, such as the American Thyroid Association and the Endocrine Society, have recommended treatment of hyperthyroidism during the first trimester of pregnancy with PTU to reduce the risk of teratogenicity to the fetus but recommended switching to MMI for the remainder of pregnancy to minimize unnecessary PTU exposure for the mother [1, 2].

Notably, these recommendations were initially formed in response to studies linking MMI (but not PTU) exposure to a variety of birth defects. However, emerging evidence now suggests that PTU may not be as safe to fetal development as previously believed and may actually pose an increased risk of malformations as well [4, 8–10]. Preliminary data suggest that the congenital anomalies associated with PTU appear to be rare, less severe, and of a different spectrum compared to those associated with MMI and as such may be easily overlooked. Accordingly, there has been a call for more reports of possible cases of PTU-associated birth defects to better characterize this condition [8]. Herein, we report the first case of congenital bands resulting in intestinal malrotation in a neonatal girl following PTU exposure in early pregnancy.

## 2. Case Presentation

A 17-day-old girl presented to hospital with irritability, decreased appetite, and repeated bilious vomiting for a day. Examination revealed a tender and distended abdomen. A subsequent computed tomography scan of the abdomen with contrast revealed the presence of a high-grade small bowel obstruction with multiple dilated, fluid-filled loops of bowel in the proximal ileum, characterized by muscosal hypoenhancement. There also appeared to be a reversal of the relationship between the superior mesenteric artery and superior mesenteric vein, suggestive of an underlying rotational abnormality.

An emergency laparotomy was then performed. Upon entering the abdomen, the cecum and ascending colon were found to be loose without any obvious lateral attachments in keeping with a rotational abnormality. Further exploration revealed a necrotic, closed loop obstruction at the distal jejunum. Using gentle traction, this twisted segment was exteriorized and several congenital bands were noted. Ligation of the bands was performed and the bowel was detorted. The segment of necrotic bowel was then resected and a primary end-to-end anastomosis was performed. The remaining bowel was examined and no other anatomical abnormalities were found. The postoperative course was uneventful.

The patient is the first child of nonconsanguineous Sri Lankan parents, born at term by elective cesarean section, and was initially discharged home two days after birth in stable condition without any perinatal complications. Incidentally, a small (3 mm) ventricular septal defect was discovered on routine newborn examination and managed expectantly. She was initially breastfed and remained well until she eventually presented with a small bowel obstruction on day 17 of life, as described. Prior to this, she had no surgeries.

Notably, during pregnancy, the child's mother, who was 29 years old, was treated for hyperthyroidism. She was diagnosed with Graves' disease 5 months before pregnancy and was initially treated with PTU 100 mg p.o. t.i.d. The dosage was reduced to 50 mg p.o. t.i.d. a month before conception; she continued on this same dose at conception and for the duration of the first trimester until the 14th week of gestation. Afterwards, MMI 5 mg p.o. q.d. was prescribed. She was clinically and biochemically euthyroid at the time of conception and thyroid function tests remained normal for the entire pregnancy. Immediately after birth, the patient's thyroid function was monitored closely because of her maternal history of Graves' disease. On the newborn screen (collected on the second day of life), her thyroid stimulating hormone (TSH) level was modestly elevated at 41 mIU/L. After two weeks, her TSH level remained elevated at 27.84 mIU/L but with a normal free T4 of 19.8 pmol/L. Although she remained well (and clinically euthyroid), levothyroxine replacement was initiated as a precaution with subsequent normalization of thyroid function tests soon thereafter.

There were no other significant maternal illnesses or medication exposures during pregnancy. The child had no other past medical history. There was no family history of any significant congenital birth defects.

## 3. Discussion

Concerns of potential teratogenicity of antithyroid drugs were initially raised in 1972 when the first report of aplasia cutis was noted in children born to mothers treated with MMI during pregnancy [11]. Since then, numerous reports and several large studies have emerged, confirming this association, as well as noting a number of other related birth defects (i.e., choanal atresia, esophageal atresia, and omphalocele), now collectively referred to as “methimazole embryopathy” [4–6]. Up until recently though, PTU was widely believed to pose no major threat to fetal development and was therefore recommended as the preferred therapy during the first trimester of pregnancy [1, 2]. However, this notion was challenged by a large Danish cohort study, which reported a higher prevalence of congenital malformations of the face, neck, and urinary systems in children exposed to PTU in early pregnancy [4]. When compared to the birth defects associated with MMI exposure, however, those associated with PTU appeared to largely involve different organ systems, tended to be less severe, and sometimes remained undiagnosed for longer periods of time [8].

Indeed, both MMI and PTU freely cross the placenta and likely pose the greatest risk to fetal development during the first trimester of pregnancy (i.e., during the critical period of organogenesis) [3]. A recent review of 91 case reports of potential birth defects associated with antithyroid drug exposure* in utero* found that, with the exception of only two cases, nearly all the reports claimed antithyroid drug exposure between the 6th week and 10th week of gestation [9]. While the timing of medication exposure appears to be crucial, there has been no detectable association between the dose of antithyroid drug and the subsequent risk of malformation [6, 9]. Therefore, the most important considerations in identifying potential cases of PTU-associated birth defects are exposure to PTU during early pregnancy as well as biological plausibility.

We propose that congenital bands may potentially represent a rare yet serious complication of PTU exposure in early pregnancy. While most cases of intestinal obstruction in the pediatric population are from postoperative adhesions or stenosis, other causes should be considered in those without prior history of abdominal surgery [12]. Uncommonly, intestinal obstruction may be secondary to embryological remnants of the vitelline vessels, omphalomesenteric ducts, or mesourachus; these are typically recognized by their anatomical location [13]. Rarely, obstruction may be caused by anomalous congenital bands without apparent embryogenic origin [14–17]. While their etiology is not well understood, it has been suggested that these fibrous bands of tissue arise from a mesenteric anomaly occurring around the 4th week of gestation and are later associated with malrotation, anomalous intestinal fixation, and obstruction [14, 17]. In our case, we also found evidence of several anomalous congenital bands associated with an intestinal rotational abnormality resulting in complete obstruction, therefore raising the suspicion of an early mesenteric developmental abnormality. To date, no teratogen has yet been implicated in the development of congenital bands. Although hypothyroidism may rarely result in intestinal pseudoobstruction (i.e., paralytic ileus) [18], this did not appear to be a contributing factor in our patient, as she was euthyroid at the time and had a demonstrable anatomic reason for her obstruction.

Of note, our patient was also incidentally found to have a small ventricular septal defect shortly after birth. Normally, the major septa of the heart are formed by the 5th week of fetal development. Incomplete closure of the interventricular septum may result in an isolated ventricular septal defect [19]. This condition is common and is estimated to be present in up to 5% of all newborns, and the vast majority close spontaneously without intervention [20]. Accordingly, multiple large studies have reported that the prevalence of ventricular septal defects among children exposed to PTU appears similar to those never exposed to any antithyroid drugs* in utero* [4, 7]. As such, it is unclear whether the presence of an incidental ventricular septal defect in our patient had any causal relationship with her prior exposure to PTU in early pregnancy at all.

It should be pointed out that thyrotoxicosis itself may be teratogenic [21]. Indeed, critics have raised concern that most of the previous large-scale studies showing an association between antithyroid drugs and congenital malformations have not consistently reported on maternal thyroid function [22–24]. As such, some have proposed that poorly controlled hyperthyroidism may be the underlying reason for the increased rate of congenital malformations rather than treatment itself [25]. However, the striking differences in the spectrum of birth defects associated with MMI compared to PTU argue that these congenital anomalies are more likely mediated by antithyroid drug exposure rather than abnormal maternal thyroid hormone levels [4]. In our case, specifically, we can confirm that maternal thyroid function was checked at least once each trimester and was normal at the time of conception and throughout the duration of the pregnancy.

The potential teratogenicity of PTU remains to be elucidated. While preliminary reports have suggested that most cases are mild in nature and are restricted to preauricular and urinary system malformations [4, 8, 9], careful consideration should also be given to other manifestations of disease that may potentially be more severe and possibly involve other organ systems too. Congenital bands may potentially represent a rare yet serious complication of PTU exposure in early pregnancy. More reports are needed to further support this association. This case report serves to alert clinicians of a potential new teratogenic association of PTU, thus prompting further confirmatory reports and future definitive studies.
